# Supplementary material for: Ultrafast nonthermal electron transfer at plasmonic interfaces
Source: Nat Commun. 2025 Nov 21;16:10410. doi: 10.1038/s41467-025-66640-9 (PMC12644476; doi:10.1038/s41467-025-66640-9)
Supplement: Supplementary file 1 — Supplementary Information [file 41467_2025_66640_MOESM1_ESM.pdf]

Supplementary Information

Ultrafast nonthermal electron transfer at plasmonic interfaces

Yuying Gao[1,2,#,*], Jonathan Diederich[1,3,4,#], Yuxin Xie[2], Qianhong Zhu[2], Christian Höhn[1], Karsten Harbauer[1], Fengtao Fan[2], Can Li[2], Roel van de Krol[1,3], Dennis Friedrich[1,*]

[1]*Institute for Solar Fuels, Helmholtz-Zentrum Berlin für Materialien und Energie GmbH, Berlin 14109, Germany;*

[2]*State Key Laboratory of Catalysis, Dalian National Laboratory for Clean Energy, Dalian Institute of Chemical Physics, Chinese Academy of Sciences, Dalian 116023, China;*

[3]*Institut für Chemie, Technische Universität Berlin, Berlin 10623, Germany;*

[4]*Department of Chemistry, Princeton University, Princeton 08544, USA;*

[#]*These authors contributed equally: Yuying Gao, Jonathan Diederich.*

*E-mail: yuying.gao@helmholtz-berlin.de; friedrich@helmholtz-berlin.de

Table of Contents

Supplementary Notes 1-10

Supplementary Figures 1-26

Supplementary References

Supplementary Notes

Supplementary Note 1. Ballistic transport velocity

The ballistic transport velocity of hot carriers can be described by:

$$E = \frac{1}{2} m_e^* v_{max}^2 \qquad (1)$$

where E is the energy vs E_F of an excited electron, m_e^* is the effective mass of the electron, and v_{max} is its ballistic velocity. The velocity v_{max} is estimated to be e.g. 8.9×10^5 m/s for 2.3 eV VIS photon energy. For electron transport from Au nanoparticles to GaN to occur within the laser pulse duration (here 40 fs), a minimum velocity of 1.5×10^5 m/s is required. Since this value is significantly lower than the maximum velocity v_{max}, it provides evidence that ballistic transport is a viable mechanism within the temporal constraints of the laser pulse.

Supplementary Note 2. Simulation of electron energy distribution

Monte Carlo simulations were employed to model the ultrafast temporal evolution of the photoexcited electron energy distribution by iteratively integrating multiple processes, including scattering, transport, and recombination. The simulation begins with an initial electron energy distribution derived from experimental two-photon photoemission (2PPE) data at 0 fs delay time for Au film/GaN system. The contribution of interface states at a kinetic energy of 1.62 eV (see details in Figure 2e) was subtracted using a Gaussian function to isolate the initial energetic profile. This profile represents the energy distribution of hot electrons generated in Au nanostructures following SPR excitation, before interfacial charge injection and significant scattering processes. This result is consistent with recent theoretical calculations in plasmonic nanostructures[1, 2]. The 2PPE spectrum at 0 ± 10 fs is processed and aligned relative to the Fermi level (E_F). The cross-correlation (CC) data from the VIS-UV overlap on

Cu (111) is utilized as the instrument response function. Scattering, relaxation, recombination, and potential electron injection into the GaN conduction band are tracked across discrete time steps to capture the ultrafast dynamics. Functions describing electron lifetimes, scattering probabilities, and energy losses are defined based on the electron energy above E_F. The electron lifetime (τ_e) depends on energy and is expressed as[1] $\tau_e(E) = \tau_0 \frac{E_F^2}{(E-E_F)^2}$, here τ_0 is a material-dependent constant[3]. The scattering probabilities $P_{scatter}(E)$ is given by $P_{scatter}(E) = 1 - e^{-\frac{\Delta t}{\tau_e(E)}}$, with Δt representing the simulation time step. The energy loss for each electron is randomly sampled from a uniform distribution ranging from 0 to ΔE, producing an array of corresponding energy loss values to serve as input for the subsequent simulation step. The injection function for hot electrons is nearly a step function of their energy. The injection probability is defined as zero for energies below the Schottky barrier height and transitions to being energy-dependent for energies slightly above the CBM of GaN[4]. For simplicity, we assumed that the momentum distribution of hot electrons at the interface satisfies the momentum requirements for interfacial charge injection. This assumption is justified by the fact that the initial momentum of the electrons is predominantly aligned along the interface normal under p-polarized light excitation as the electric field enhancement dominates the anisotropic momentum distribution of hot electrons[5, 6]. This approach enables the simulation to record the total number of electrons injected into the GaN conduction band at each pump-probe time step. Recombination probability for each electron is calculated based on its energy. Electrons are marked as recombined if a random value falls below their respective probabilities, and the total number of recombined counts is determined. At specified intervals, the energy distribution and scattering events of the remaining electrons are recorded to monitor electron dynamics. Enhanced recombination rates are applied to electrons near E_F to account for possible interface-state-mediated recombination, which allows us to better reproduce the experimental results of energy distribution dynamics (Supplementary Fig. 15b). Electrons with

energies below E_F are removed in the simulation to reflect their reintegration into the Fermi Sea. Finally, this simulation framework provides the detailed tracking of electron energies, scattering counts, and electron populations at each time step.

Supplementary Note 3. Excluding the Auger recombination mechanism in hot electron relaxation

In our experiment, the 2PPE intensities at zero delay time exhibit a linear dependence on laser intensity for Au NP/GaN. This linear behavior rules out significant contributions from multiphoton processes under the given pump-probe conditions, as nonlinear photothermal and photothermoelectric effects would be expected to show superlinear and sublinear power dependencies, respectively[7].

The dynamics of hot carriers are inherently complex, involving various recombination mechanisms, such as electron-hole recombination, defect trapping, and Auger recombination[8]. Accounting for these possible recombination mechanisms, the rate of change of the excited electron concentration (n) can be described as follows[8]:

$$\frac{\mathrm{d}n}{\mathrm{d}t} = -k_1 n - k_2 n^2 - k_3 n^3 \qquad (2)$$

where k_1 denotes the recombination rate related to defect trapping, k_2 denotes the bimolecular electron-hole recombination rate, and k_3 denotes the non-radiative Auger-recombination rate. Varying pump power intensity shows that hot carrier dynamics are independent of the laser excitation intensity, allowing us to rule out the significant contribution of Auger recombination processes in Au NP/GaN.

Supplementary Note 4: Estimation of charge injection efficiency

Since the photoemission intensity is directly proportional to the electron population, the total number of photoexcited carriers can be estimated from the energy distribution spectra, a method widely used in time-resolved photoemission spectroscopy studies[9, 10]. The total number of plasmon-induced electrons can thus be estimated using the following expression:

$$N = \int_{0} I(E)dE \qquad (3)$$

where $I(E)$ is the electron energy spectrum. As previously demonstrated, the energy distribution spectrum of photoemitted electrons above the Schottky barrier at 0 fs delay reflects the contribution of electrons transferred into GaN, while the signals below the Schottky barrier are attributed to electrons remaining within the Au nanoparticles. Consequently, the total number of transferred electrons (N_t) can be determined using the following expression:

$$N_t = \int_{E \geq 1.1} I(E)dE \qquad (4)$$

Based on the measured energy distribution spectrum at 0 fs, the efficiency of hot electron transfer is calculated to be approximately 31%.

Supplementary Note 5. Identifying the interface states for both Au film/GaN and Au NP/GaN samples

For Au film/GaN samples, emission peaks associated with interface states at a kinetic energy of 1.62 eV exhibit time-dependence (Figure 2e in the Results), indicating that the emission process involves one VIS and one UV photon. Given that excitation at positive delay times occurs via VIS (2.38 eV), and emission occurs via UV photons (4.33 eV), the occupied interfacial state is located at ~0.65 eV below the Fermi level.

In the Au NP/GaN system, interface state emission becomes more clearly observable for VIS pump photon energies exceeding 2.48 eV (Supplementary Fig. 18). The feature around kinetic

energies of 0.3 eV can be probed in more detail by increasing pump photon energies, as this reduces the spectral overlap between UV 1PPE and VIS 2PPE from the Fermi level. This signal is independent of delay time, and its intensity exhibits a quadratic relationship with VIS pump intensity (see Supplementary Fig. 19), indicating that the observed emission from interface states occurs via two VIS photons. Fitting the 2PPE spectra with a composite Voigt function reveals two interfacial states on Au NP/GaN, located at 0.53 eV and 0.75 eV below the Fermi level.

Supplementary Note 6. Fitting temporal evolution of 2PPE spectra to extract lifetimes

Photoelectron population dynamics for a range of kinetic energies were modelled using an exponential decay, convolved with the Gaussian response function of initial photoexcitation. The hot electron decay for Au NP/GaN was therefore fitted with the following equation[11]:

$$f(t) = \mathrm{A} \cdot e^{\left(-\frac{(t-t_0)^2}{2w^2}\right)} * e^{-\frac{t}{\tau}} \qquad (5)$$

where t is the delay time, t_0 is the pump-probe temporal overlap, w is the temporal width of the laser pulse (about 40 fs), τ is the decay time, and A is a proportionality factor.

For Au film/GaN samples, high-energy electron thermalization results in a significant increase in the population of low-energy photoelectrons within 100 fs. Thus, a different formula is used for the Au film/GaN sample to decouple signal growth resulting from high-energy photoelectron relaxation. The electronic lifetimes were determined according to the following expression:

$$\frac{df(t)}{dt} = \mathrm{A} \cdot e^{\left(-\frac{(t-t_0)^2}{2w^2}\right)} - \frac{f(t)}{\tau} + B \cdot \left(1 - e^{-\frac{t}{\tau_0}}\right) \qquad (6)$$

where the first term describes the Gaussian time profile of the laser pulse, the second term represents the hot electron decay process with a time constant τ, and the third term accounts

for the rise in photoelectrons due to the relaxation of high-energy electrons, denoted by a time constant τ_0. A rise time constant of 22 fs determined from the lifetime of high-energy electrons was used in the fitting process.

Supplementary Note 7. Estimation of electron back-transfer time

Photoexcited electrons from the Au nanoparticles that are injected into the GaN substrate rapidly lose most of their energy through electron-phonon scattering. These electrons then transfer back to the Au nanoparticles to compensate for the charge initially injected into the GaN substrate. As a result, the relaxation time for low-energy electrons in the Au NP/GaN system is longer compared to the Au film/GaN sample, where no charge transfer across the interface occurs and thus no back-transfer takes place. This observation is consistent with the spectral features observed in the TR-2PPE data (Figure 3c). The electron back-transfer time (τ_b) in Au NP/GaN is estimated by the difference in low-energy electron lifetime between Au NP/GaN and Au film/GaN samples (positive delay times in TR-2PPE), and is given by:

$$\tau_b^{-1} = \tau_{NP}^{-1} - \tau_{film}^{-1} \qquad (7)$$

where τ_{NP} and τ_{film} are the electron lifetimes of Au NP/GaN and Au film/GaN samples respectively. Based on the measured lifetime of low-energy electrons that may be influenced by back-injected electrons, we estimate the electron back-transfer time τ_b in Au NP/GaN to be ~120 fs. This result is consistent with similar observations in the literature for Au/WSe_2, where the back-injection process occurs on timescales of around 250-300 fs[11]. Hot electrons in Au nanoparticles with energies of 1.1–2.34 eV above E_F can be injected into the GaN conduction band. After injection, they relax toward the CBM via LO phonon scattering. While the scattering time in GaN is fast (~18 fs), electrons that have not fully relaxed after multiple scattering events may back-inject into Au on a longer timescale (~120 fs), leading to delayed thermalization of lower-energy carriers. The absence of discernible features associated with

interface states in the energy-dependent lifetime analysis further suggests that their contribution to back-injection is negligible within experimental uncertainty.

Supplementary Note 8. Calculation of the electron–phonon scattering time in GaN

The electron–phonon scattering time (τ_0) in GaN may be determined via the following equation[12]:

$$\frac{1}{\tau_0} = \frac{e^2 \omega_{LO}}{2\pi\hbar} \sqrt{\frac{m^*}{2\hbar\omega_{LO}}} \left(\frac{1}{\varepsilon_\infty} - \frac{1}{\varepsilon_s}\right) \qquad (8)$$

where m^* is the effective mass, e is the electron charge, $\hbar\omega_{LO}$= 92 meV is the optical phonon energy of GaN, and ε_∞ =5.35ε_0 and ε_s = 9.5ε_0 are the high frequency and static dielectric constants respectively. Based on this, τ_0 was determined to be 18 fs in GaN.

Supplementary Note 9. Extracting hot carrier electron temperatures

When the energy of hot carriers relative to the Fermi level (E_F) is significantly larger than $k_B T$, the energetic distribution of hot carriers can be characterized by an elevated carrier temperature, which can significantly exceed the lattice temperature. To experimentally determine electron temperatures at a range of delay times, the measured TR-2PPE spectra for kinetic energies larger than 1.1 eV were fitted using the Fermi-Dirac distribution[8]:

$$I(E) = I_0(E) \frac{1}{e^{\frac{(E-E_F)}{k_B T_e}} + 1} \qquad (9)$$

where $I(E)$ is the 2PPE intensity for a given hot carrier energy, $I_0(E)$ is the proportionality factor, which represents the photoemission cross-section, E is the excited electron energy relative to the Fermi level, and T_e is the electron temperature.

Supplementary Figures

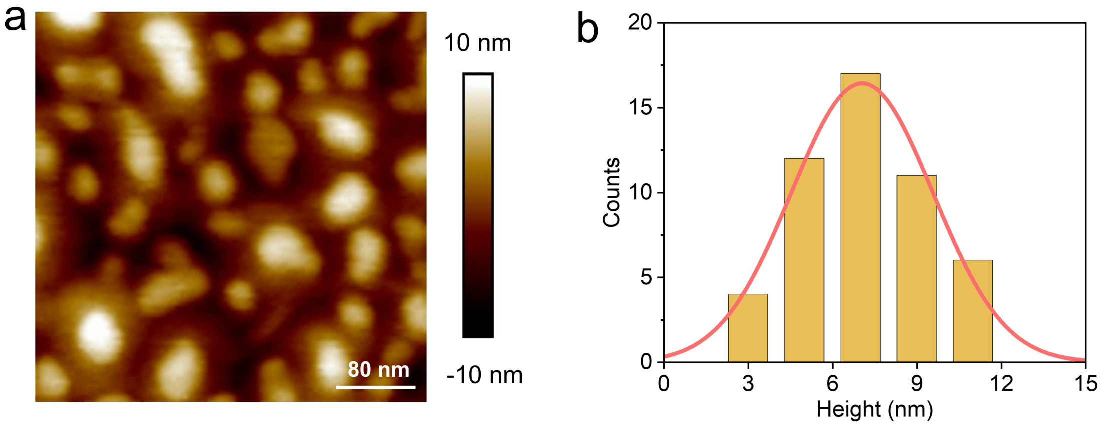

Supplementary Figure 1. Surface characterization of the Au NP/GaN sample. (a) AFM images of Au nanoparticles on a GaN crystalline surface. (b) Height distribution of Au nanoparticles and Gaussian fit to the height distribution; the average size of Au nanoparticles is 7.0 ± 0.2 nm.

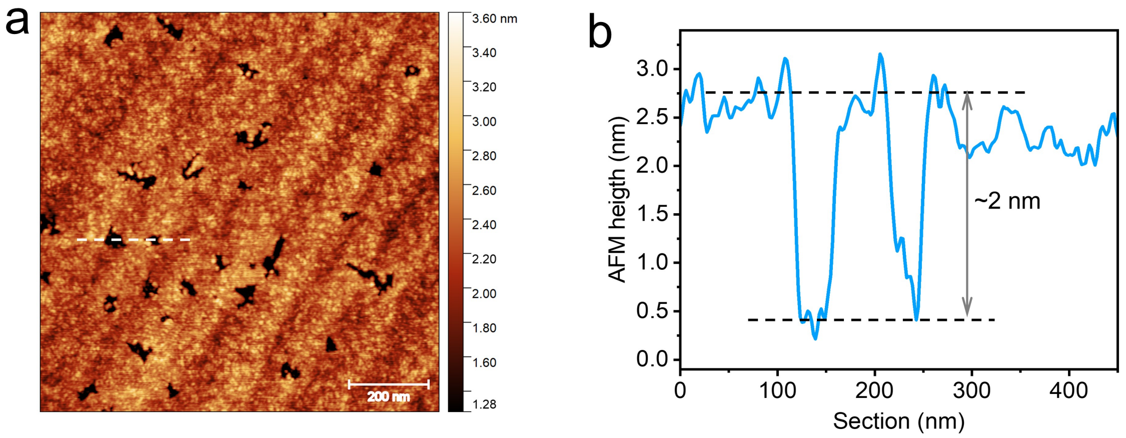

Supplementary Figure 2. Surface characterization of the Au film/GaN sample. (a) AFM morphology of an Au film/GaN sample. (b) Example height distribution profile of the Au film/GaN sample, taken along the dashed white line in a. The thickness of Au film is ~2 nm.

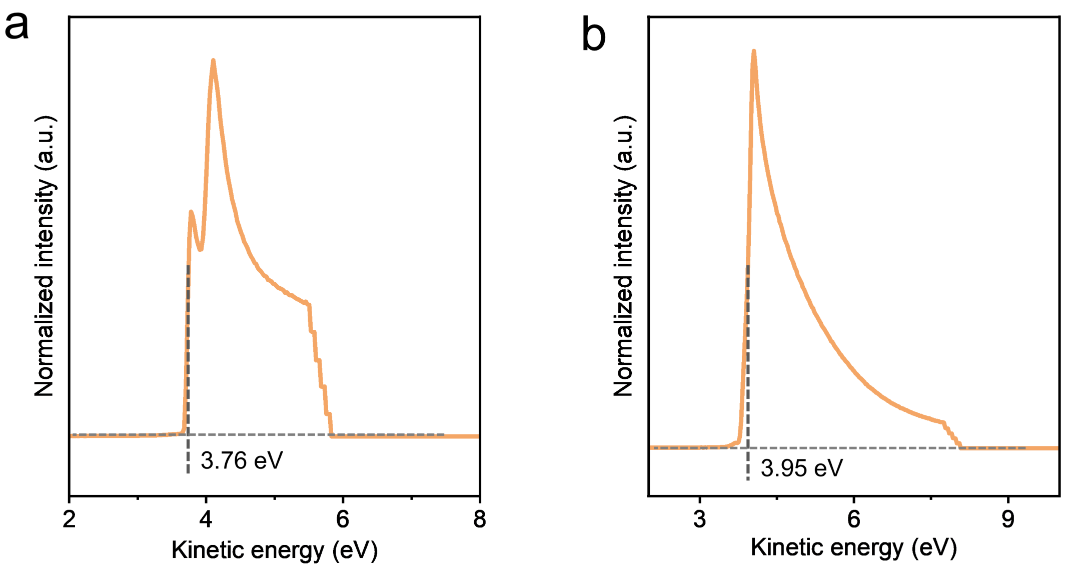

Supplementary Figure 3. Determination of the work function of the GaN wafer. Work function edges in UPS measured on (a) as-prepared GaN crystal and (b) GaN after Ar^{+} sputtering for 10 mins.

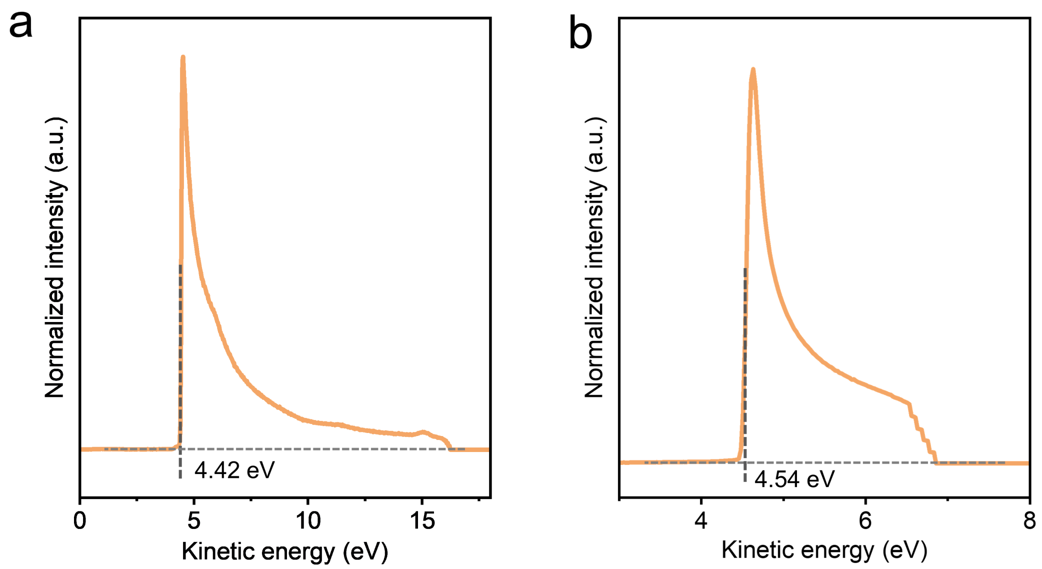

Supplementary Figure 4. Determination of the work function of the samples. Work function edges in UPS of the (a) Au NP/GaN sample and (b) Au film/GaN sample.

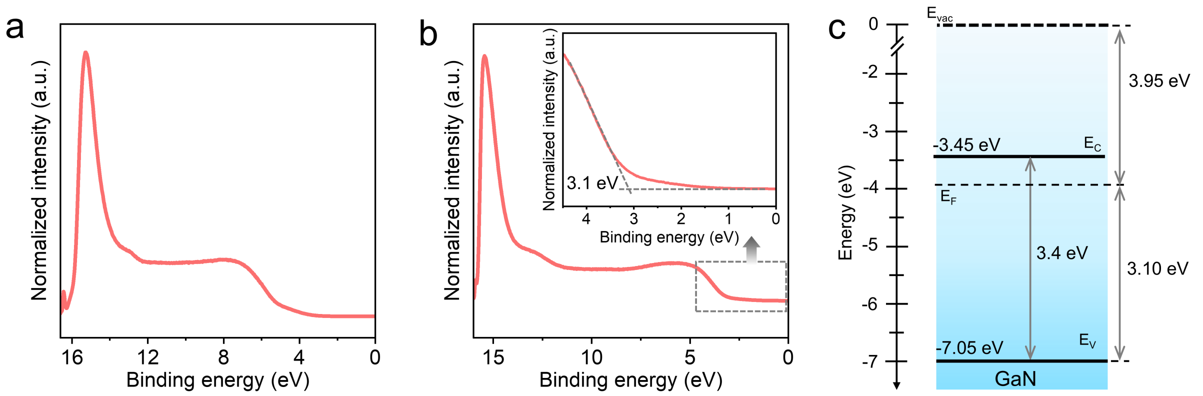

Supplementary Figure 5. Energy band distribution of the GaN surface. (a,b) Valence band spectra for the GaN surface before sputtering (a) and after sputtering (b). The inset in b is an enlarged view of the data points. The energetic position of the VBM is determined by linear extrapolation of the low binding energy edge of the valence band, the GaN surface VBM is 3.1 eV below E_F. (c) Energy band distribution of the GaN sample determined by UV-Vis absorption spectroscopy and UPS measurements.

The comparison in UPS for the GaN (0001) surface before and after cleaning treatment provides direct evidence of the surface structure. For the as-prepared GaN in air, the VBM is located 4.8 eV below the Fermi level, indicating the formation of a thin Ga_2O_3 film on the GaN (0001) surface[13]. After three cycles of Ar ion sputtering and annealing in the UHV chamber, the valence band maximum (VBM) position is significantly shifted, now at 3.1 eV below the Fermi level. Meanwhile, the conduction band minimum (CBM) is located 0.3 eV above the Fermi level, indicating the characteristic electronic properties of an n-type GaN semiconductor[13, 14].

Based on experimental measurements of surface work function and valence-band spectroscopy, we determined an energy difference of 0.3 eV between the Fermi level and the conduction band for the uncoated GaN sample (Supplementary Figure 5c). Given an effective conduction band density of states (N_c) of 2.3×10^{18} cm^{-3} for GaN[15], the corresponding carrier density is calculated to be 1.9×10^{13} cm^{-3}, indicating that GaN is nearly intrinsic with negligible surface band bending. Due to the higher work function of gold compared to GaN, the deposition

of Au NPs induces an increase in band bending. From the observed shift in the surface conduction band edge, we estimate a band bending of 0.16 eV following Au deposition.

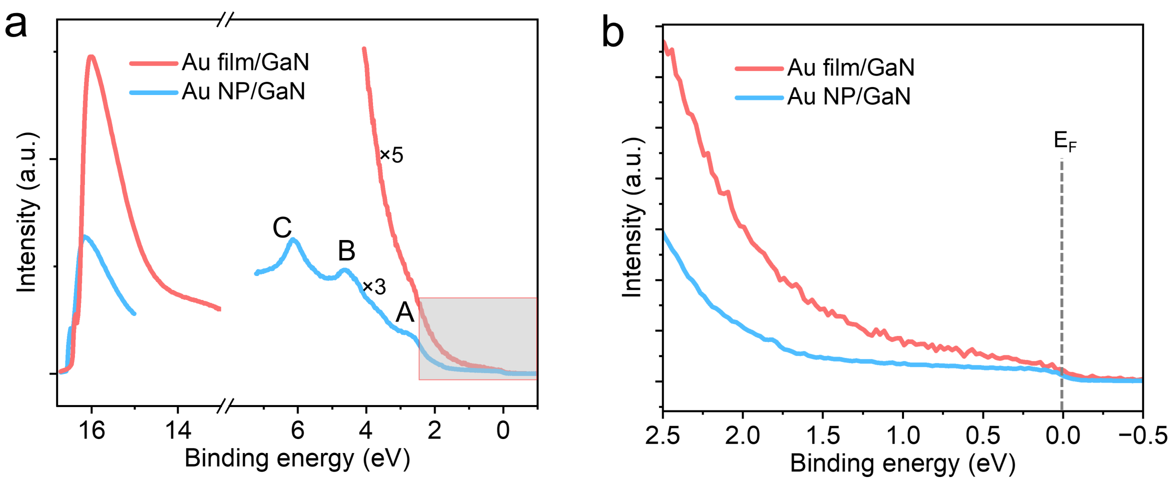

Supplementary Figure 6. Valence band spectra of the samples. (a) UPS of the Au NP/GaN and Au film/GaN surfaces. (b) An enlarged view of the data points indicated in grey in a for both the Au NP/GaN and Au film/GaN surfaces.

Three distinct peaks appear in the near-VBM UPS for Au NP/GaN. As marked in Supplementary Fig. 6a, the peaks A and C are located at 2.6 and 6.1 eV respectively, and are attributed to the Au 5d electron[16]. Peak B, located at 4.6 eV, can be attributed to the N 2*p* and Ga 4*p* electronic states. Because of the effect of the additional density of states around the VBM after Au deposition[17], the surface VBM of Au/GaN can be calculated using the expression $Ga3d_{GaN}–VBM_{GaN}=Ga3d_{Au/GaN}–VBM_{Au/GaN}$, where $Ga3d_{GaN}$ and $Ga3d_{Au/GaN}$ are the respective binding energies of the Ga 3d core level for the bare GaN and Au/GaN samples. Based on the Ga 3d peak position in XPS, the VBM of the Au NP/GaN sample is estimated to be 2.27 eV below the Fermi level.

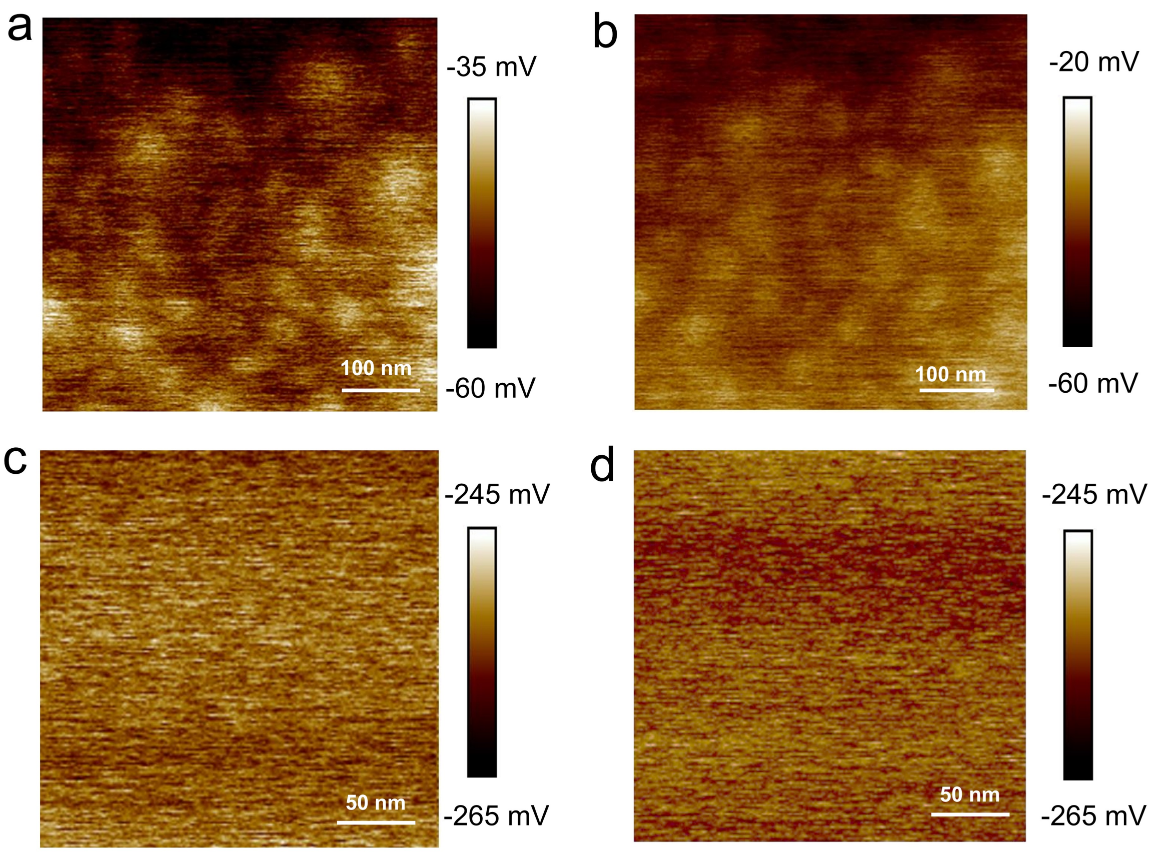

Supplementary Figure 7. (a,b) Surface potential images of Au NP/GaN in dark conditions (a) and under 520 nm light illumination (b). (c,d) Surface potential images of Au film/GaN in dark conditions (c) and under 520 nm light illumination (d).

The spatially non-uniform distribution of the measured surface potential originates from the distribution of Au nanoparticles on the surface. Since the work function of gold is higher than that of the GaN surface, a higher surface potential is generated at the locations of the Au nanoparticles.

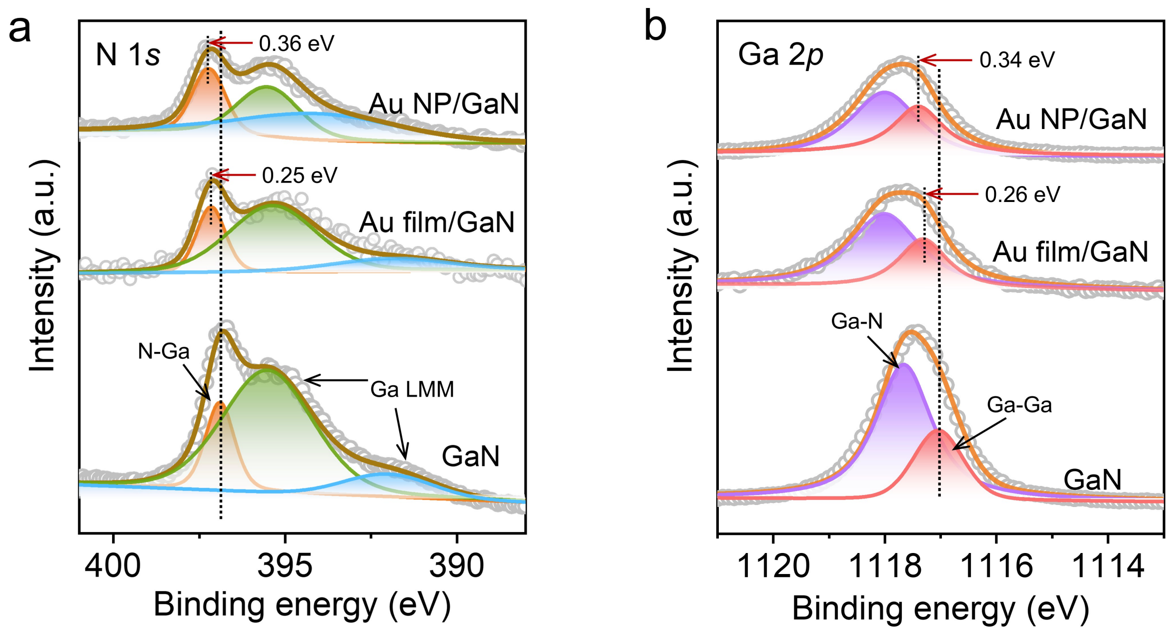

Supplementary Figure 8. XPS spectra of the Au NP/GaN and Au film/GaN samples. (a) High-resolution XPS spectra of the N 1*s* peak for bare GaN, Au film/GaN and Au NP/GaN samples. (b) High-resolution XP spectra of the Ga 2*p* peak for GaN, Au film/GaN and Au NP/GaN samples.

The N 1*s* peaks are located at binding energies of 392.0 eV and 395.5 eV, originating from Ga LMM Auger electrons. In contrast with pristine GaN, the N1*s* peak of Au film/GaN samples originating from N–Ga is shifted to higher binding energies, suggesting the transfer of electrons from GaN to Au after Au thin film deposition. After the annealing treatment to form Au NP, the energetic position of the N-Ga bonding state was further shifted by about 0.36 eV toward higher binding energies compared with the bare GaN sample, indicating strong electronic interaction between the Au NP and GaN. Meanwhile, the Ga 2*p* peak on Au NP/GaN was also shifted to higher binding energies with respect to bare GaN and Au film/GaN, revealing a decrease in electron density following Au deposition and subsequent annealing treatment. The peak at a binding energy of 1117.7 eV corresponds to the Ga-N bond, and the smaller peak at 1117.1 eV corresponds to Ga-Ga bonding[18]. Notably, the absence of gallium oxide peaks at higher binding energies suggests a clean GaN surface[19]. These results confirm the effective bonding of Au NP to the GaN substrate, which is likely to facilitate interfacial charge transfer[20].

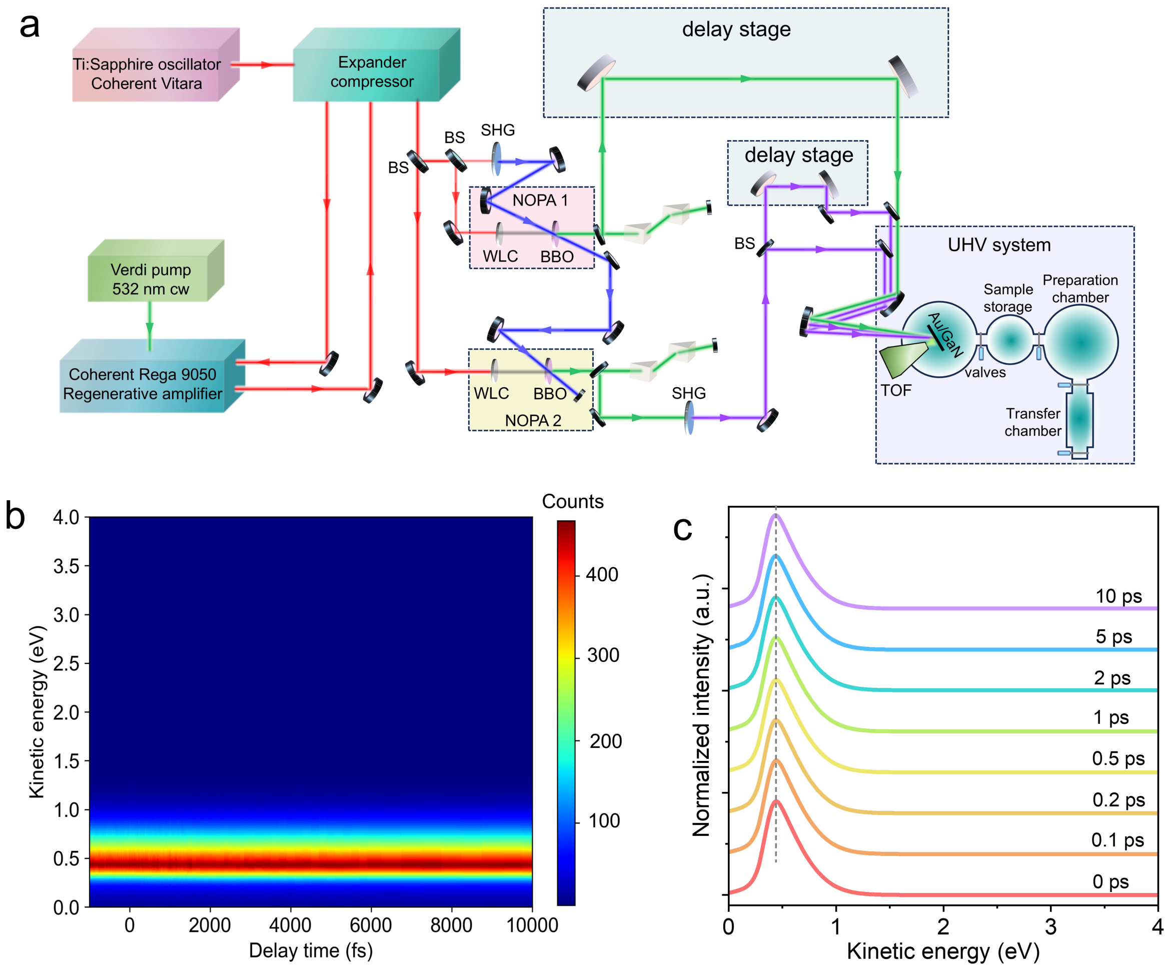

Supplementary Figure 9. TR-2PPE measurements. (a) Schematic of the set-up used to measure the TR-2PPE in UHV chamber at room temperature. The delay stage was used to control the delay time of pump-probe pulses. BS, beam splitter; BBO, β-barium borate crystal; SHG, Second Harmonic Generation; NOPA, noncollinear optical parametric amplifier; WLC, white-light continuum; TOF, time-of-flight. (b) Pseudo-colour plot of TR-2PPE spectra of the GaN (0001) surface after excitation using 2.34 eV pump and 4.49 eV probe pulses. (c) TR-2PPE kinetic energy spectra for the GaN (0001) surface for a range of pump-probe delay times.

Only a single time-independent 2PPE peak is observed, as the available laser energy is insufficient to excite GaN across its bandgap. Additionally, this result suggests that any potential intraband states do not contribute to the time-dependent 2PPE signal.

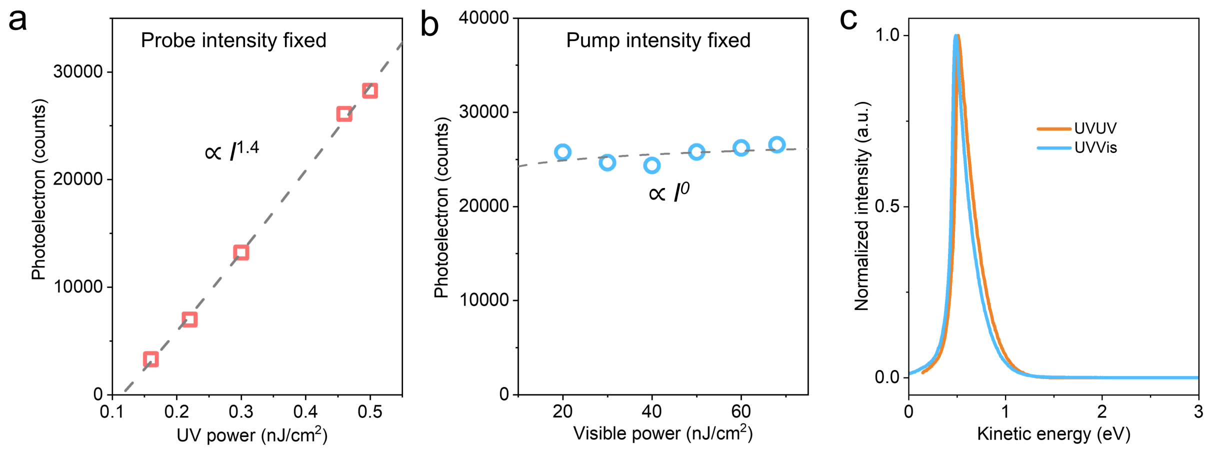

Supplementary Figure 10. Laser fluence dependence of the 2PPE signal. (a) Detected photoelectron counts as a function of UV (286 nm) pulse fluence, with the VIS (530 nm) fluence fixed. The fitted exponent of 1.4 suggests that, in addition to emission involving a single UV photon, two-photon UV emission also occurs. This implies that a portion of the observed emission may originate from a partially occupied surface state, which could be partially populated through the interband excitation of GaN by UV light. (b) Photoelectron counts as a function of VIS (530 nm) pulse fluence, with the UV (286 nm) fluence fixed. The gray dashed lines are power-law fits, suggesting a UV 1PPE process in the GaN sample, with emission occurring from around the surface Fermi level. (c) Comparison of photoelectron spectra under different pump-probe conditions for the bare GaN sample. The UV-UV pump-probe data was collected with a pump of 286 nm (0.13 nJ) and a probe of 286 nm (0.06 nJ). The VIS - UV data used a pump of 286 nm (0.13 nJ) and a probe of 530 nm (13 nJ). The similar observed spectral features provide additional evidence that visible light illumination has no substantial impact on the photoelectron emission signal.

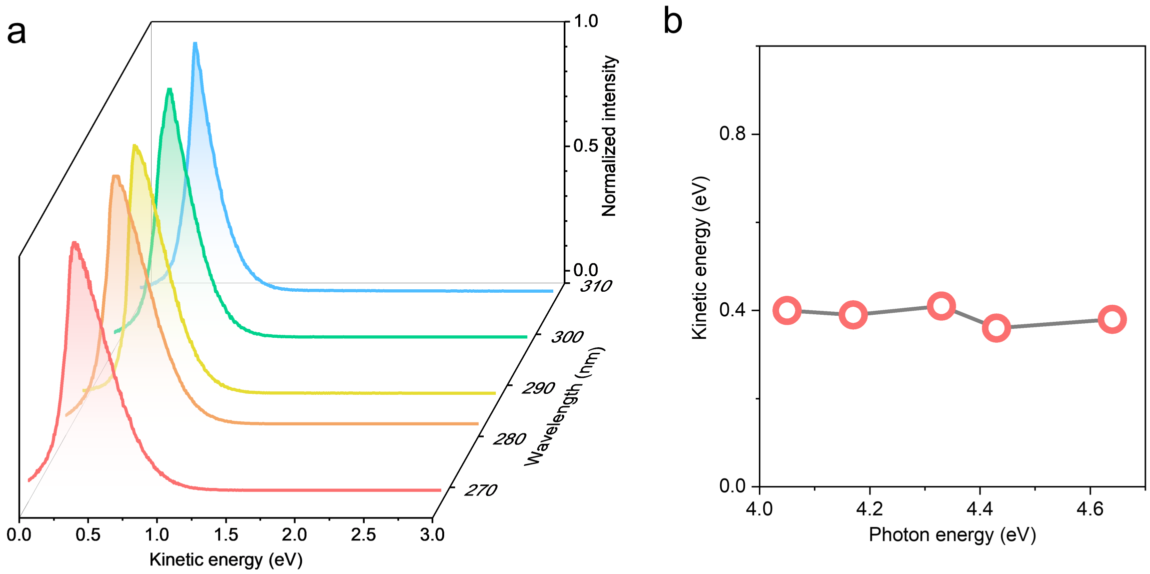

Supplementary Figure 11. Pump-energy dependence of 2PPE spectra. (a) Normalized one-colour 2PPE spectra of the bare GaN (0001) surface with the variation of excitation wavelengths between 260 nm and 310 nm. (b) Peak kinetic energy as a function of photon energy for bare GaN. The 2PPE emission peak is independent of the pump energy, indicating that it originates from surface states above the vacuum level.

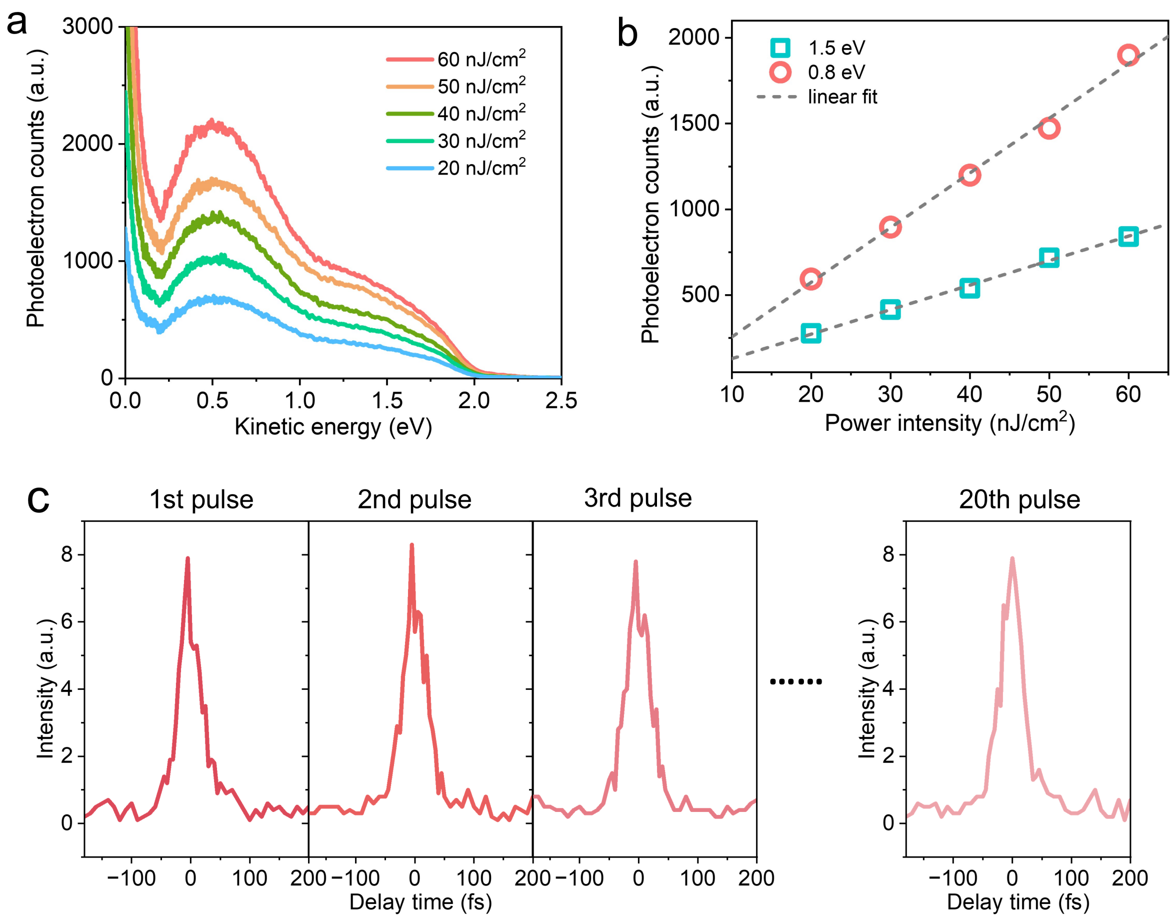

Supplementary Figure 12. Laser fluence dependence of TR-2PPE spectra. (a) 2PPE spectra of Au NP/GaN under different VIS pump (2.34 eV) fluences, at fixed UV probe (4.33 eV) fluences. (b) Photoelectron counts at 0.8 and 1.5 eV kinetic energy, each shown as a function of pump power intensity for the Au NP/GaN sample. The dashed grey line represents a linear fit. (c) Time-dependent 2PPE signal for Au NP/GaN excited at a pump energy of 2.34 eV over repeated pulse cycles, suggesting the robustness of the interface under prolonged excitation.

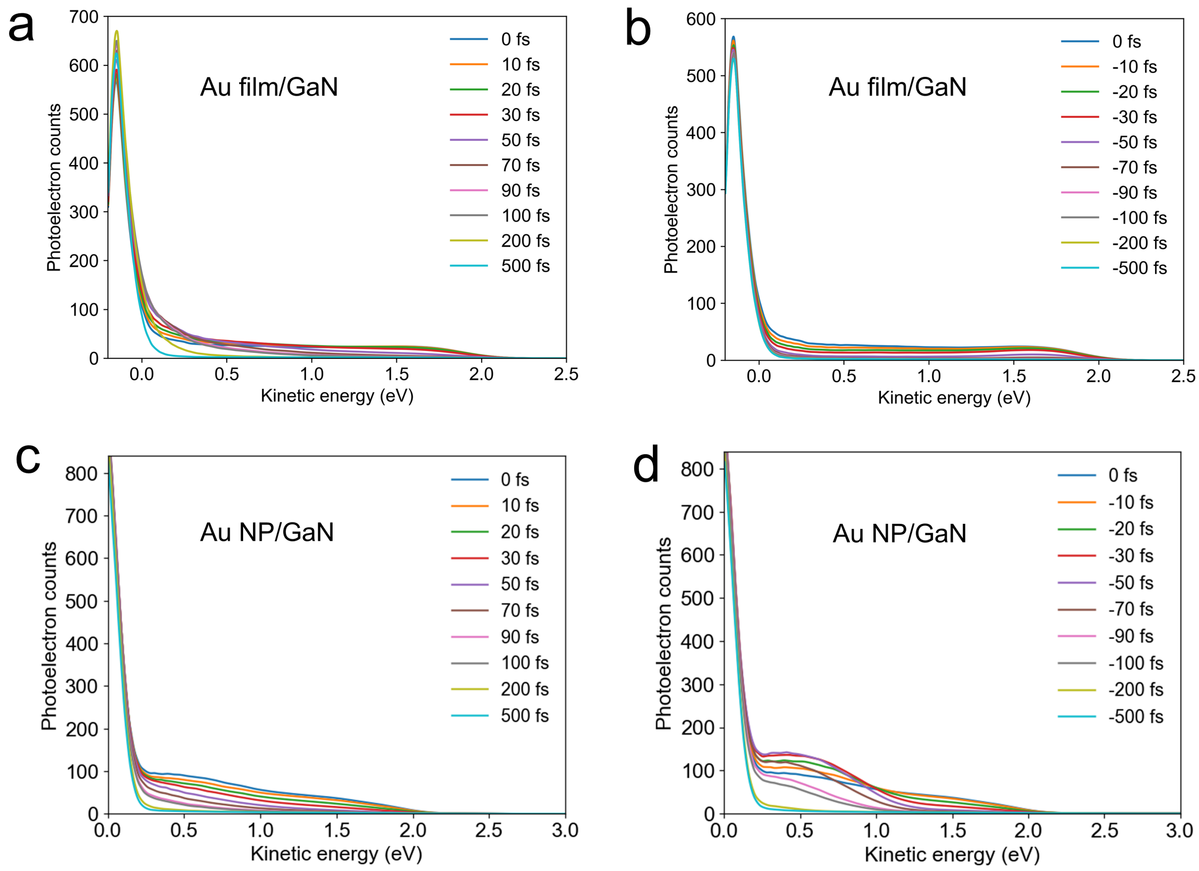

Supplementary Figure 13. TR-2PPE spectra of the Au film/GaN and Au NP/GaN samples. (a,b) 2PPE spectra of Au film/GaN at positive pump-probe delay times (a) and at negative delay times (b) using 2.34 eV VIS and 4.49 eV UV pulses. (c,d) 2PPE spectra of Au NP/GaN at positive (c) and at negative delay times (d), using 2.34 eV VIS and 4.34 eV UV pulses. At positive delay times, the samples are excited by the VIS pump, and photoelectrons are emitted by the UV probe. At negative delay times, the order is reversed: the UV pulse induces the initial electronic excitation, while the VIS pulse subsequently releases the excited electrons into the vacuum.

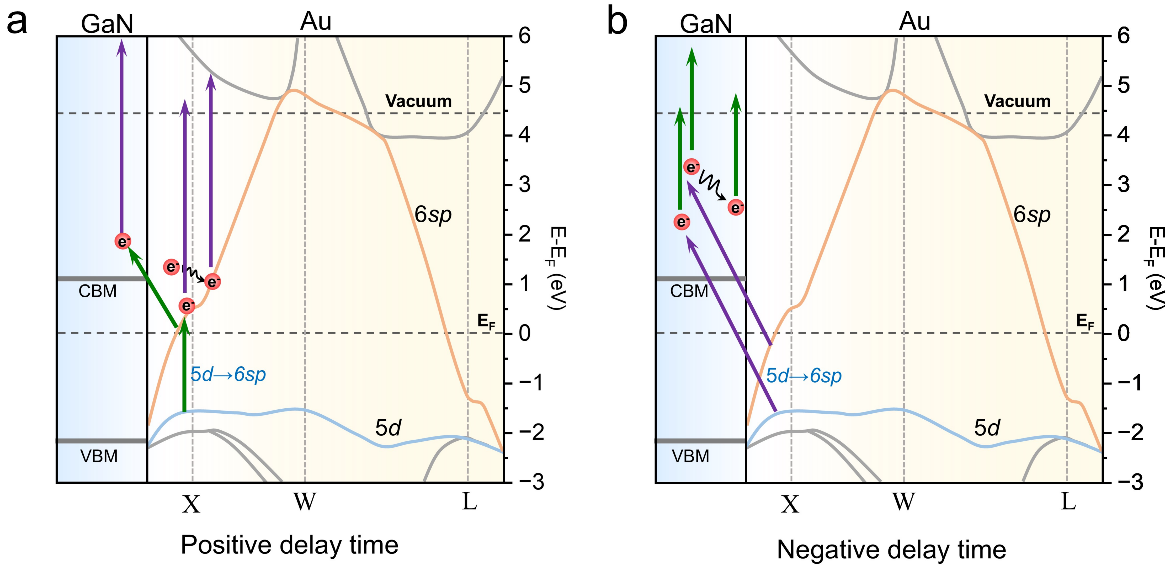

Supplementary Figure 14. Schematic of TR-2PPE processes at positive and negative delay times. (a) Schematic illustration of Au NP/GaN excited by VIS and photoemitted by UV pulses (positive delay time). (b) Schematic illustration of Au NP/GaN excited by UV and photoemitted by VIS pulses (negative delay time).

At positive delay times, the visible light serves as the pump to excite the sample. Only high-energy electrons generated from intraband excitation have sufficient energy to overcome the Schottky barrier and inject into the GaN. However, electrons generated from interband excitation are distributed near the Fermi level and cannot undergo interfacial charge transfer. In contrast, at negative delay times, UV pulse acts as the pump, both intraband and interband excitation-generated hot electrons can inject into the conduction band of GaN. Therefore, the observed signal at negative delay times originates from GaN, resulting in a broader energy distribution.

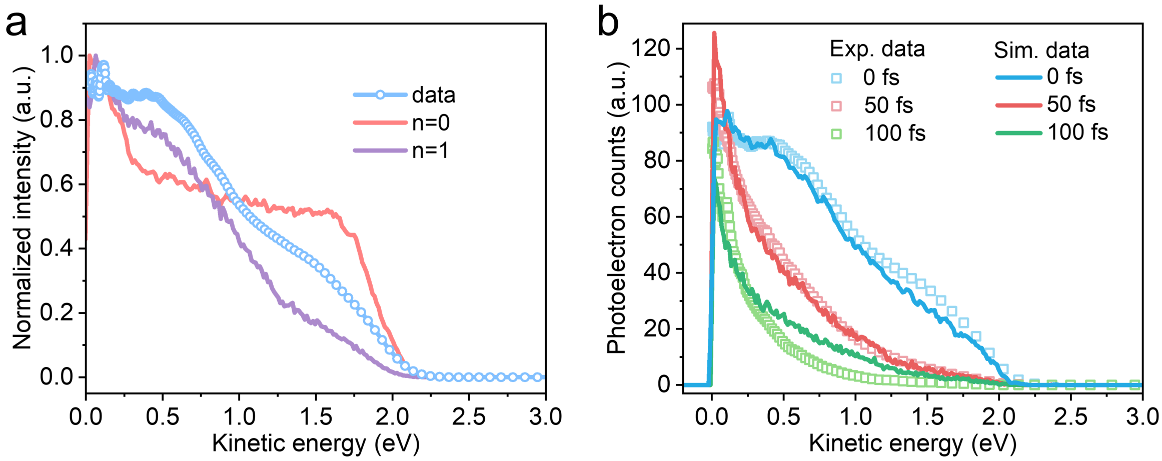

Supplementary Figure 15. Theoretical simulation of energetic distribution following SPR excitation. (a) Experimental data (open blue circle) and simulations (lines) of electron energy distribution curve for Au NP/GaN at 0 fs delay. The red line represents the simulated initial electron energy distribution without scattering interactions (n=0) and the subsequent charge transport process upon SPR excitation. The purple line illustrates the simulated initial electron energy distribution with interfacial charge injection, accounting for scattering events (n=1). (b) Simulated and experimental data of electron energy population at different delay times. The simulation details can be found in Supplementary Note 2. The experimental signals were determined from Au NPs/GaN sample (Figure 2d).

Ultrafast scattering events result in a reduction in the population of high-energy electrons while increasing the population of low-energy electrons. Additionally, interfacial electron injection contributes to the decrease in the 2PPE intensity of high-energy electrons. Thus, we consider both the effects of scattering events and interfacial injection on the electron energy distribution at 0 fs delay time in simulation. Even under the assumption that each electron undergoes only a single scattering event during charge transport (purple line in Supplementary Fig. 15a), the intensity of high-energy electron populations in the simulation remains lower than the experimental results. This indicates that the charge transfer process is less affected by electron-electron scattering, and the electrons transferred to GaN maintain a relatively high-energy distribution, indicative of quasi-ballistic transport regime.

We monitored the temporal evolution of the electron energy distribution upon SPR excitation of Au nanoparticles, incorporating the combined effects of electron scattering, charge injection, and charge recombination. The theoretical results exhibit strong quantitative agreement with the experimental signals (Supplementary Fig. 15b). This remarkable consistency is achieved under the assumption that each scattering event results in a 55% energy loss. This suggests that electron-electron scattering dominates the energy relaxation process of hot electrons, with each collision halving the energy of the hot electrons. The results suggest that low-energy electrons generated after scattering may lack sufficient energy to overcome the interfacial Schottky barrier. However, the strong interaction within the present configuration facilitates ultrafast interfacial electron transfer occurring before substantial electron scattering, thereby enhancing charge separation and collection efficiencies. Thus, by engineering interfacial electronic structures, plasmon excitation in metal nanostructures could enable highly efficient operation of photodetectors and photovoltaic devices within the true ballistic transport regime, even when the plasmon excitation wavelength does not match the electronic bandgap of semiconductor. This mechanism represents a paradigm shift from the previously established plasmon-induced interfacial charge-transfer transition pathway, where direct charge generation in semiconductors arise from damped plasmon bands in metal/semiconductor systems[21, 22].

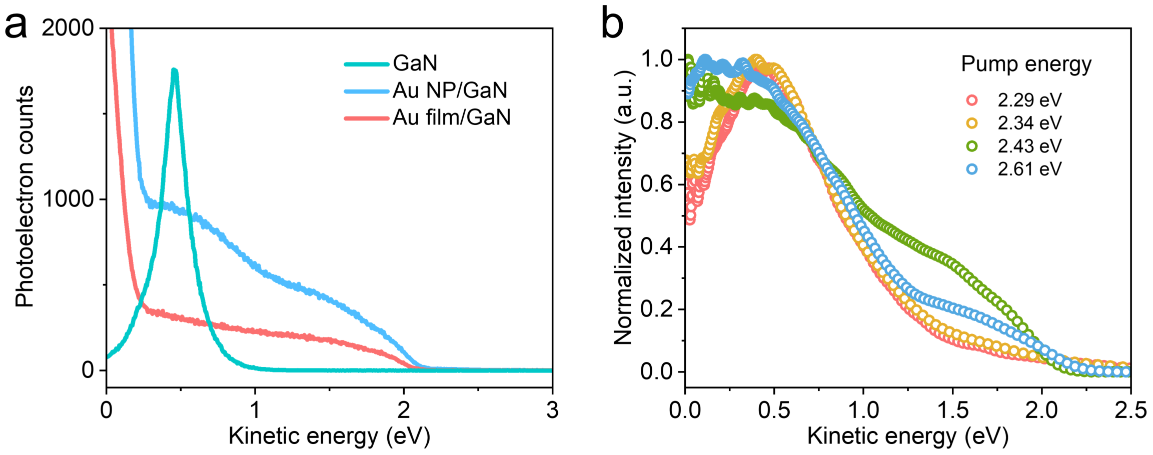

Supplementary Figure 16. Comparison of TR-2PPE spectra at different pump energies. (a) Comparison of 2PPE spectra for bare GaN, Au NP/GaN and Au film/GaN samples pumped with VIS (2.43 eV, 30 nJ/cm^2) and probed by UV pulses (4.34 eV, 0.6 nJ/cm^2), spectra taken at zero delay between pump and probe. (b) Normalized 2PPE spectra of Au NP/GaN at 0 fs pump-probe delay for a range of VIS pump photon energies.

The time-independent peak of GaN around 0.45 eV cannot be observed for either the Au NP/GaN or Au film/GaN in Supplementary Fig. 16a, likely due to the suppression of GaN emission, or the passivation of surface states after Au deposition. The significant increase in signal strength at kinetic energies of less than 0.3 eV is attributed to 1PPE from the Fermi level. The 2PPE signal intensity of Au NP/GaN samples is clearly higher than that of Au film/GaN samples, indicating the enhancement of photo-induced charge generation in Au NP/GaN due to SPR effect.

Due to differences in optical alignment and laser spot size across different measurement sequences, photoemission counts vary between different excitation energies. To isolate the effect of pump photon energy on the energetic distribution of excited electrons, we normalize the 2PPE spectra at the interband transition photoemission peak, situated around a kinetic energy of 0.35 eV, as the interband transition is less affected by the SPR effect. We found that the proportion of high-energy electrons (kinetic energy>1.0 eV) initially increases with photon energy (Supplementary Fig. 16b), reaching a maximum at SPR resonant excitation (2.43 eV),

and then subsequently decreasing. This result demonstrates that the surface plasmon effects of Au NP can enhance the generation and transfer of high-energy electrons at the interface.

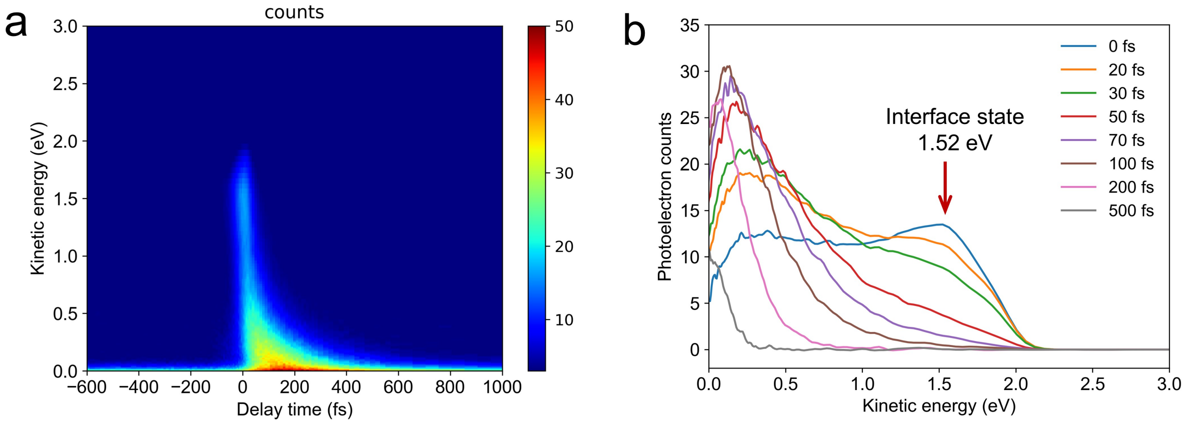

Supplementary Figure 17. Representative pseudo-color plot of TR-2PPE spectra of Au film/GaN. (a) TR-2PPE transient spectrum pumped by 2.21 eV VIS and probed by 4.53 eV UV pulses for Au film/GaN sample. (b) Time-independent 2PPE spectra for Au film/GaN sample at different delay times by subtracting a reference data slice at 3000 fs delay from each spectrum.

In contrast to the Au film/GaN sample pumped at 2.34 eV (Figure 2c), the spectral feature related to the interface state between Au and GaN can be more clearly observed using a 2.21 eV VIS pump. The kinetic energy of the interface state shifts to 1.52 eV, which agrees well with results obtained at 2.34 eV VIS photon energy. Based on these results, we can determine that the interface state is positioned at 0.65 eV below the Fermi level.

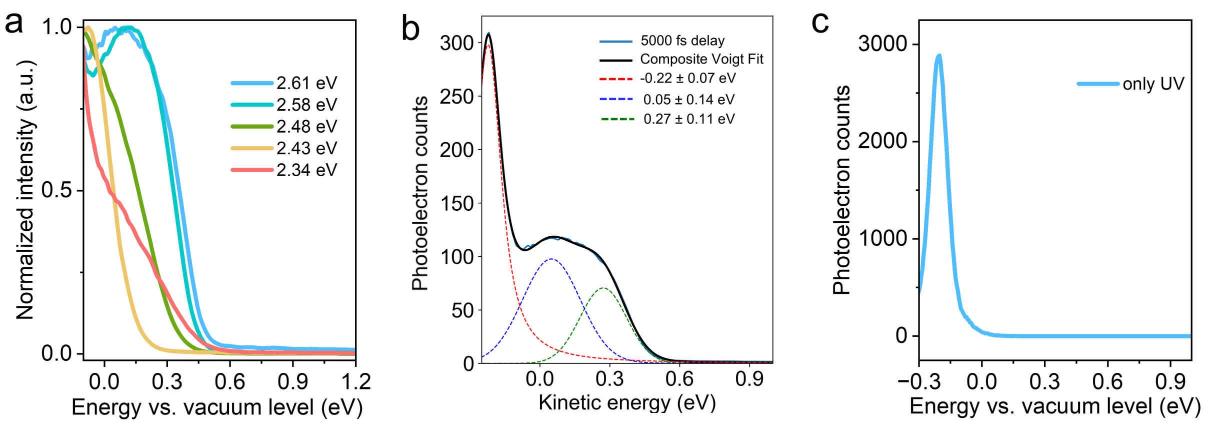

Supplementary Figure 18. TR-2PPE spectra of the Au NP/GaN. (a) Time-independent photoelectron emission spectra obtained at 5000±1000 fs pump-probe delay time for Au NP/GaN, pumped by different VIS photon energies and probed at 4.34 eV UV. The time-independent signal may be caused by one UV photon or two VIS photons. The observed dependence on VIS photon energy rules out the possibility of UV 1PPE, thereby confirming that the signal originates from the emission of interface states induced by two VIS photons. (b) Composite Voigt fit for time-independent photoelectron emission at 5000 ± 1000 fs for Au NP/GaN, excited at 2.61 eV and probed at 4.34 eV. The feature around 0.2 eV clearly represent two individual peaks, separated by ca. 0.22 eV, and is related to VIS 2PPE from around the Fermi level. (c) Photoelectron emission of Au NP/GaN with UV illumination only (4.34 eV) measured at the applied voltage of 0.5 eV. The photoemission signal can be attributed to UV 1PPE from the Fermi level. Since the work function (4.42 eV) of Au NP/GaN is greater than the energy of the UV photons (4.34 eV), the UV 1PPE signal can only be detected when a bias voltage is applied. The dramatic difference in the kinetic energy of the emitted signal for Au NP/GaN under VIS-UV excitation and individual UV excitation further confirms that the two time-independent peaks in a and b originate from VIS 2PPE of interface states.

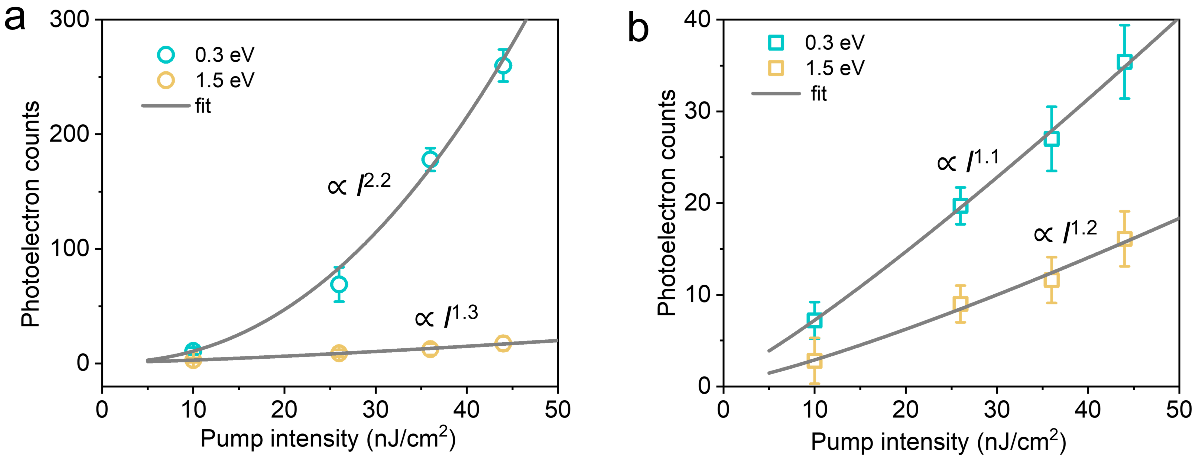

Supplementary Figure 19. Laser intensity dependence of 2PPE intensity. (a) Photoelectron counts at 0 fs delay time of the Au NP/GaN as a function of VIS pump power intensities at different kinetic energies. (b) Time-dependent photoemission of the Au NP/GaN as a function of VIS pump fluences by subtracting the background signal taken at 3 ps delay time. The grey lines are power fits.

The fitting results show that the exponent is close to 2 at low kinetic energies (0.3 eV), while it is closer to 1 at higher kinetic energies (1.5 eV). This indicates that low-energy photoemitted electrons primarily originate from VIS 2PPE processes, whereas high-energy photoelectrons are generated through the interaction of one UV photon and one VIS photon, as two VIS photons alone lack the required energy to produce high-energy photoelectrons. As anticipated, when considering only the time-dependent components of the emission, the photoemission scaling with fluence approaches a power of 1, indicating time-dependent photoemission induced by the simultaneous absorption of one VIS and one UV photon.

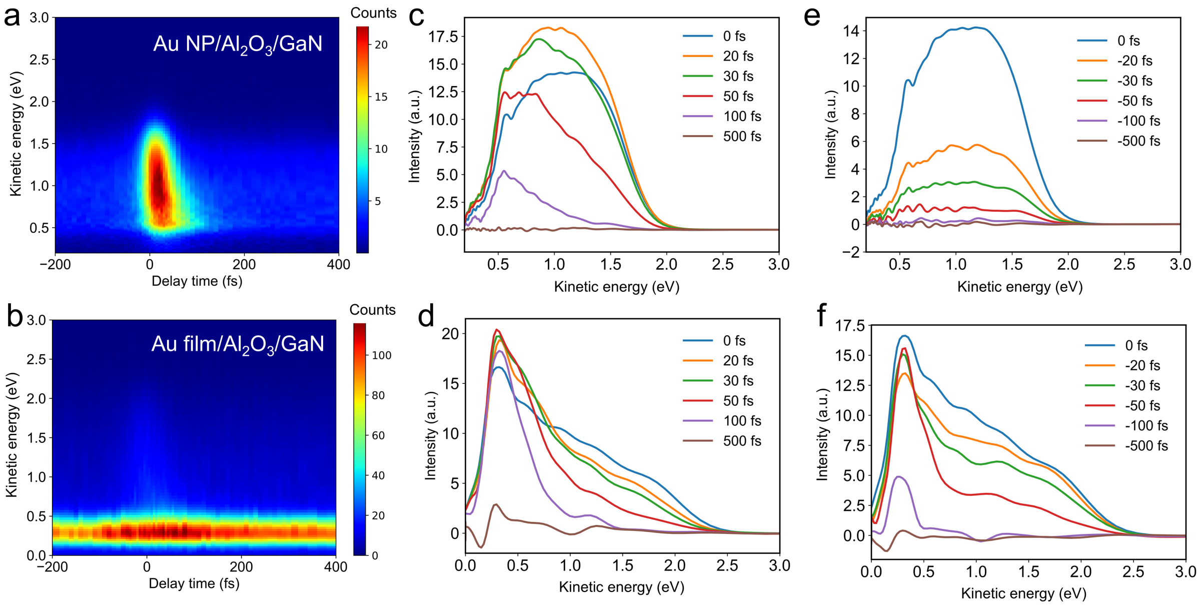

Supplementary Figure 20. (a) Representative pseudo-color plot of TR-2PPE spectra of Au NP/Al_2O_3/GaN excited at 2.25 eV and probed at 4.33 eV. (b) Representative pseudo-color plot of Au film/Al_2O_3/GaN excited at 2.25 eV and probed at 4.33 eV. (c,d) Background-subtracted 2PPE spectra of Au NP/Al_2O_3/GaN (c) and Au film/Al_2O_3/GaN (d) samples at the positive delay times. (e,f) Background-subtracted 2PPE spectra of Au NP/Al_2O_3/GaN (e) and Au film/Al_2O_3/GaN (f) samples at the negative delay times.

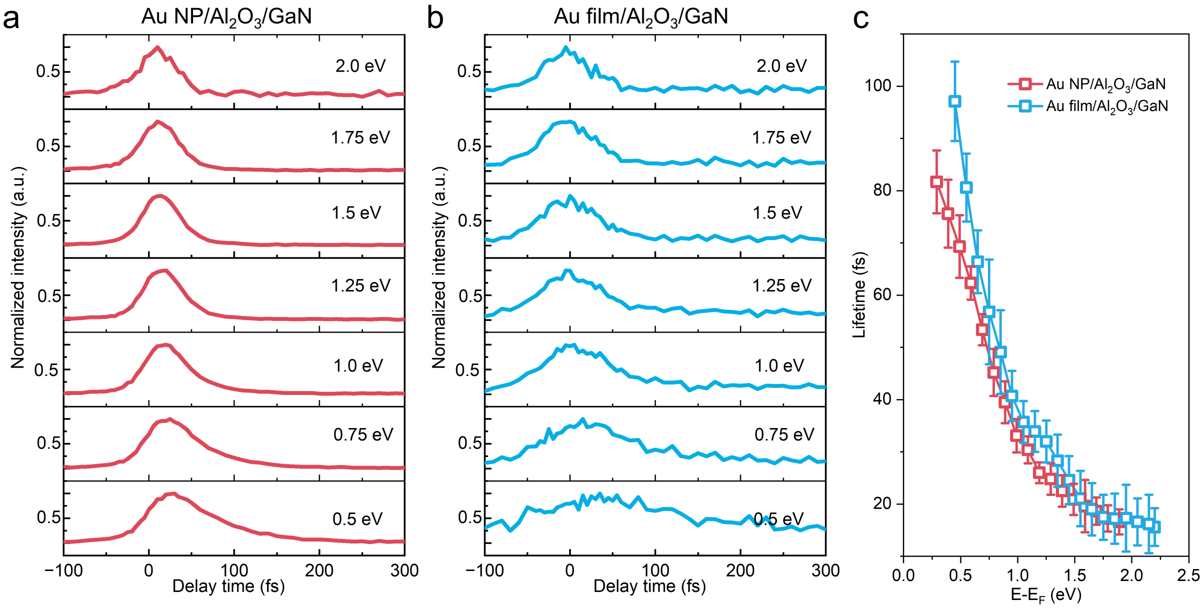

Supplementary Figure 21. (a) Energy-resolved photoelectron intensity as a function of delay time for Au NP/Al_2O_3/GaN samples excited at 2.25 eV. (b) Energy-resolved photoelectron intensity as a function of delay time for Au film/Al_2O_3/GaN excited at 2.25 eV. (c) Lifetimes of excited carriers as a function of excitation energy E-E_F for both Au NP/Al_2O_3/GaN and Au film/Al_2O_3/GaN samples.

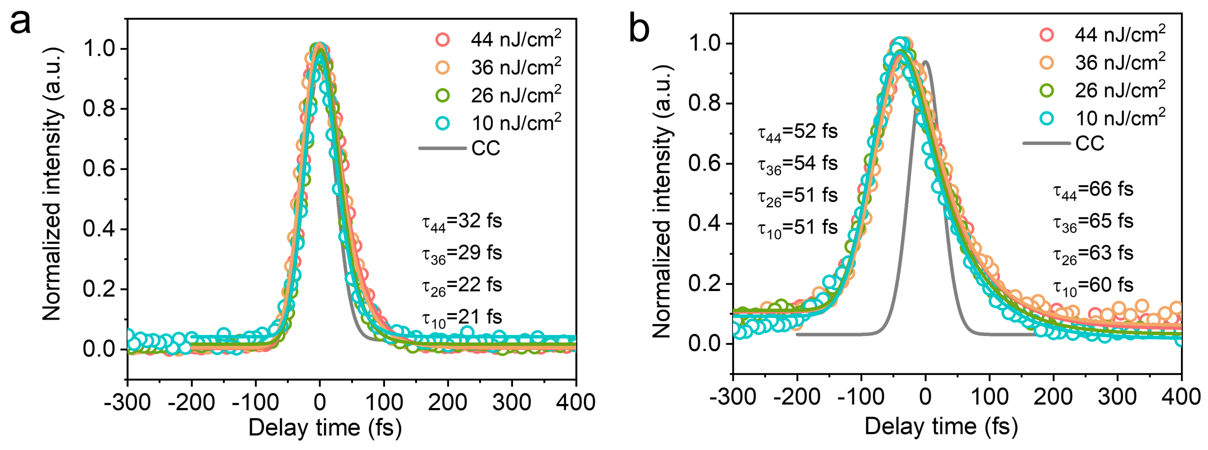

Supplementary Figure 22. Temporal evolution of the 2PPE signal dependence on pump intensity. (a,b) Normalized photoelectron intensities for the high-energy spectral region (1.8-2.3 eV) (a) and low-energy spectral region (0.2-1.0 eV) (b) of Au NP/GaN as a function of pump-probe delay times, obtained for a range of different pump intensities. The pump-probe cross correlation (CC, grey lines) measured on a Cu crystal is also shown for comparison. The decay profiles at positive delay times were fitted using an exponential function convolved with a symmetric Gaussian shape, to account for contributions from the instrument response. For the low-energy spectral range at negative delay times, decays were fitted with an exponential decay equation with a rise function, to account for high-energy electron thermalization. More details on the formulas used in fitting can be found in Supplementary Note 6.

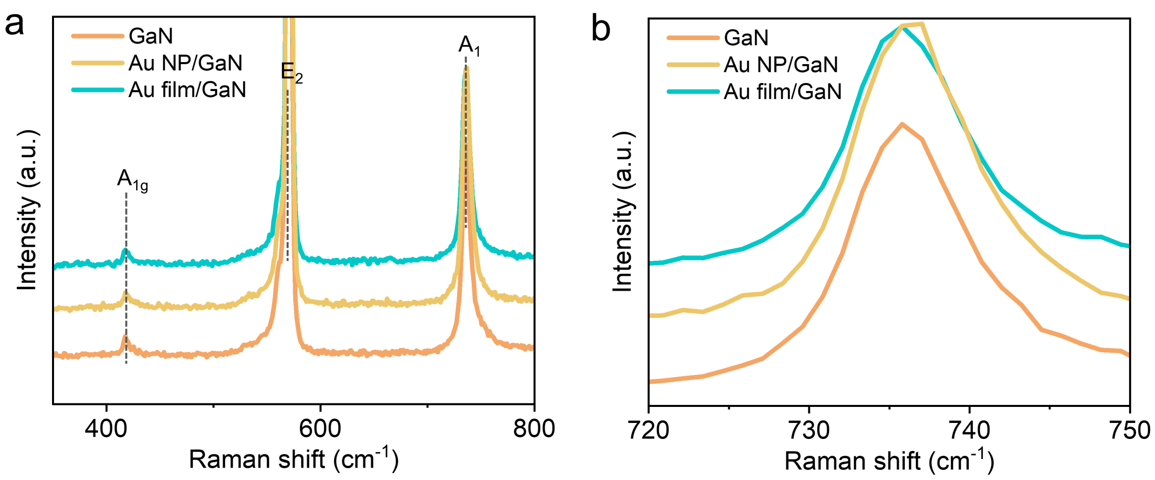

Supplementary Figure 23. Raman spectra measurements. (a) Raman spectra of bare GaN deposited on a sapphire substrate, Au NP/GaN and Au film/GaN. (b) A1 peak of Raman spectra for bare GaN, Au NP/GaN and Au film/GaN.

The Raman peaks located at 736, 569 and 417 cm^{-1} can be attributed to the A_1 (longitudinal optical, LO) phonon mode of GaN, E_2 mode of GaN and A_{1g} mode of the sapphire substrate, respectively[23]. The observation of active phonon modes on the c-plane wurtzite GaN surface proves the high quality of the single crystal. The LO phonon energy in GaN is estimated to be 91 meV, which is in excellent agreement with previous results[24]. After Au deposition, the Raman intensity was increased compared to bare GaN. Additionally, the FWHM of the A_1 peak for Au NP/GaN increased by 3 cm^{-1} with respect to bare GaN. According to the energy–time uncertainty relationship[25], the presence of Au results in the reduction of the phonon lifetime (from 0.66 ps to 0.5 ps), likely indicating a shorter electron-phonon interaction time in Au/GaN. This is caused by LO phonon-electron scattering dominating hot electron relaxation in GaN.

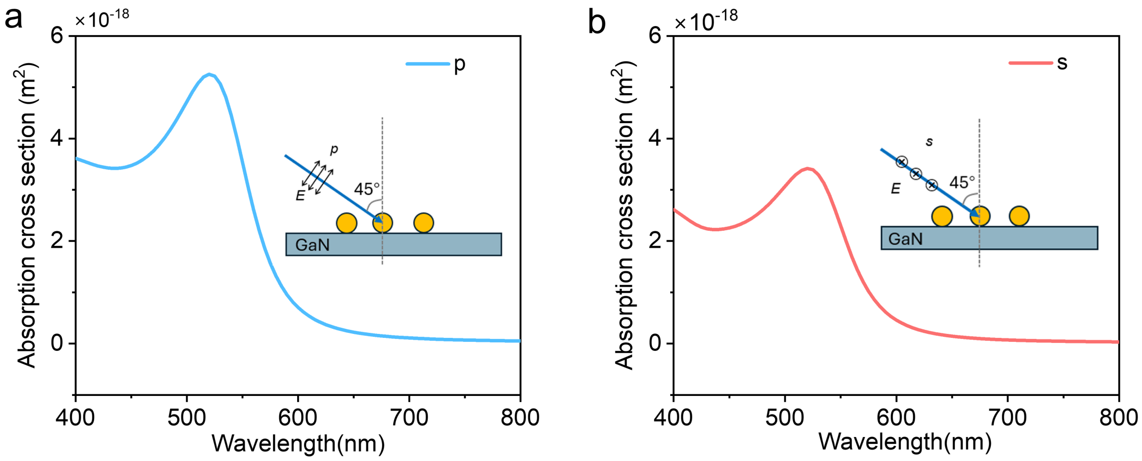

Supplementary Figure 24. Calculated absorption spectra under p- and s-polarized excitation. (a) Calculated absorption spectra of Au NP/GaN under p polarized light. (b) Calculated absorption spectra of Au NP/GaN under *s* polarized light. The inset illustrates schematically the mode of optical excitation used.

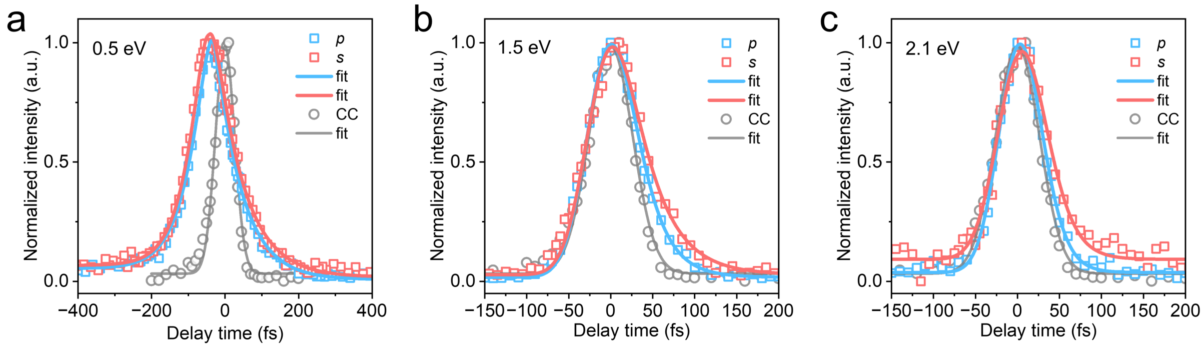

Supplementary Figure 25. Influence of polarization on electron dynamics. (a-c) Comparison of normalized 2PPE intensities as a function of pump-probe delay times for Au NP/GaN samples for *p* polarization (open blue squares) and *s* polarization (open red squares) of the VIS pump, taken at kinetic energies of 0.5 eV (a), 1.5 eV (b) and 2.1 eV (c). The solid lines are fits to the experimental data, using an exponential function convolved with a Gaussian function. The pump-probe cross correlation (CC, open grey circles) on crystalline Cu is shown for comparison. The grey curve is a fit of CC data using a Gaussian function.

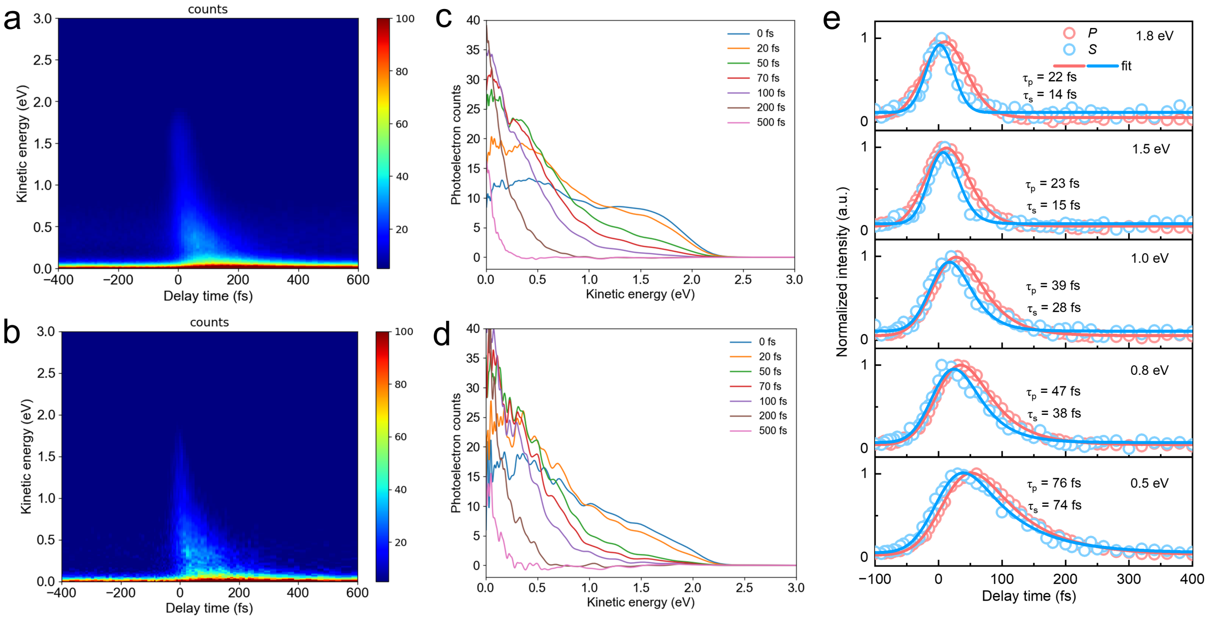

Supplementary Figure 26. Polarization dependence of TR-2PPE for the Au film/GaN. (a,b) TR-2PPE 3D transient plots of Au film/GaN samples for *p* polarization (a) and *s* polarization (b) of the VIS pump at 2.34 eV photon energy. (c,d) Corresponding 2PPE spectra at different delay times for VIS *p* polarization (c) and *s* polarization (d), with the time-independent background (taken at 3000 fs) subtracted. (e) Comparison of normalized time-dependent 2PPE intensity for VIS *p* polarization (open red circles) and *s* polarization (open blue circles) at different kinetic energies. The solid lines are fitted using an exponential model convoluted with a Gaussian response function to account the time resolution of the setup (details in Supplementary Note 6). The lifetime of hot carriers using *p*-polarized VIS pulses is longer than when using *s*-polarized VIS light for the Au film/GaN samples.

Supplementary References:

1. Schirato, A., Sanders, S.K., Zaccaria, R.P., Nordlander, P., Della Valle, G. & Alabastri, A. Quantifying Ultrafast Energy Transfer from Plasmonic Hot Carriers for Pulsed Photocatalysis on Nanostructures. *ACS Nano* **18**, 18933-18947 (2024).
2. Jermyn, A.S., Tagliabue, G., Atwater, H.A., Goddard, W., Narang, P. & Sundararaman, R. Transport of hot carriers in plasmonic nanostructures. *Phys. Rev. Mater.* **3**, 075201 (2019).
3. Brown, A.M., Sundararaman, R., Narang, P., Goddard, W.A. & Atwater, H.A. Nonradiative Plasmon Decay and Hot Carrier Dynamics: Effects of Phonons, Surfaces, and Geometry. *ACS Nano* **10**, 957-966 (2016).
4. Ng, C. et al. Hot Carrier Extraction with Plasmonic Broadband Absorbers. *ACS Nano* **10**, 4704-4711 (2016).
5. Christopher, P. & Moskovits, M. Hot Charge Carrier Transmission from Plasmonic Nanostructures. *Annu. Rev. Phys. Chem.* **68**, 379-398 (2017).
6. Li, X.H., Chou, J.B., Kwan, W.L., Elsharif, A.M. & Kim, S.G. Effect of anisotropic electron momentum distribution of surface plasmon on internal photoemission of a Schottky hot carrier device. *Opt. Express* **25**, A264-A273 (2017).
7. Chen, Y.Z., Li, Y.J., Zhao, Y.D., Zhou, H.Z. & Zhu, H.M. Highly efficient hot electron harvesting from graphene before electron-hole thermalization. *Sci. Adv.* **5** (2019).
8. Fu, J.H. et al. Hot carrier cooling mechanisms in halide perovskites. *Nat. Commun.* **8**, 1300 (2017).
9. Rohde, G. et al. Ultrafast Formation of a Fermi-Dirac Distributed Electron Gas. *Phys. Rev. Lett.* **121**, 256401 (2018).
10. Gierz, I. et al. Tracking Primary Thermalization Events in Graphene with Photoemission at Extreme Time Scales. *Phys. Rev. Lett.* **115**, 086803 (2015).
11. Pincelli, T. et al. Observation of Multi-Directional Energy Transfer in a Hybrid Plasmonic-Excitonic Nanostructure. *Adv. Mater.* **35** (2023).
12. Yang, Y. et al. Observation of a hot-phonon bottleneck in lead-iodide perovskites. *Nat. Photonics* **10**, 53-59 (2016).
13. Grodzicki, M., Mazur, P., Zuber, S., Brona, J. & Ciszewski, A. Oxidation of GaN(0001) by low-energy ion bombardment. *Appl. Surf. Sci.* **304**, 20-23 (2014).
14. Tracy, K.M., Mecouch, W. J., Davis, R. F., Preparation and characterization of atomically clean, stoichiometric surfaces of n- and p-type GaN(0001). *J. Appl. Phys.* **94**, 3163-3172 (2003).
15. Madelung, O. Semiconductors: Group IV Elements and III-V Compounds (Data in Science and Technology). (1991).
16. Zou, C.W. et al. Initial interface study of Au deposition on GaN(0001). *Physica B* **370**, 287-293 (2005).
17. Grodzicki, M. Properties of Bare and Thin-Film-Covered GaN(0001) Surfaces. *Coatings* **11** (2021).
18. Kumar, M., Kumar, A., Thapa, S.B., Christiansen, S. & Singh, R. XPS study of triangular GaN nano/micro-needles grown by MOCVD technique. *Mater. Sci. Eng. B-Adv.* **186**, 89-93 (2014).
19. Hashizume, T. Effects of Mg accumulation on chemical and electronic properties of Mg-doped-type GaN surface. *J. Appl. Phys.* **94**, 431-436 (2003).
20. He, B. et al. Strong Interactions between Au Nanoparticles and $BiVO_4$ Photoanode Boosts Hole Extraction for Photoelectrochemical Water Splitting. *Angew. Chem. Int. Ed.* **63**, e202402435 (2024).
21. Wu, K., Chen, J., McBride, J.R. & Lian, T. Efficient hot-electron transfer by a plasmon-induced interfacial charge-transfer transition. *Science* **349**, 632-635 (2015).

22. Tan, S., Argondizzo, A., Ren, J., Liu, L., Zhao, J. & Petek, H. Plasmonic coupling at a metal/semiconductor interface. *Nat. Photonics* **11**, 806-812 (2017).
23. Zeng, Y. et al. Raman Analysis of E (High) and A (LO) Phonon to the Stress-Free GaN Grown on Sputtered AlN/Graphene Buffer Layer. *Appl. Sci-Basel* **10** (2020).
24. Park, K., Mohamed, A., Dutta, M., Stroscio, M.A. & Bayram, C. Electron Scattering via Interface Optical Phonons with High Group Velocity in Wurtzite GaN-based Quantum Well Heterostructure. *Sci. Rep-Uk* **8** (2018).
25. Hofstetter, D., Beck, H. & Bour, D.P. Ultra-Short Lifetime of Intersubband Electrons in Resonance to GaN-Based LO-Phonons at 92 meV. *Photonics-Basel* **10** (2023).
